# Apolipoprotein M Gene (*APOM*) Polymorphism Modifies Metabolic and Disease Traits in Type 2 Diabetes

**DOI:** 10.1371/journal.pone.0017324

**Published:** 2011-02-24

**Authors:** Jun-Wei Zhou, Stephen K. W. Tsui, Maggie C. Y. Ng, Hua Geng, Sai-Kam Li, Wing-Yee So, Ronald C. Ma, Ying Wang, Qian Tao, Zhen-Yu Chen, Juliana C. N. Chan, Yuan-Yuan Ho

**Affiliations:** 1 Department of Biochemistry, The Chinese University of Hong Kong, Hong Kong, China; 2 Department of Pediatrics, Center for Diabetes Research, Wake Forest University Health Sciences, Winston-Salem, North Carolina, United States of America; 3 Cancer Epigenetics Laboratory, State Key Laboratory in Oncology in South China, Department of Clinical Oncology, Sir YK Pao Center for Cancer, Li Ka Shing Institute of Health Sciences, The Chinese University of Hong Kong, Hong Kong, China; 4 Department of Medicine and Therapeutics, The Chinese University of Hong Kong, Hong Kong, China; 5 Hong Kong Institute of Diabetes and Obesity, Hong Kong, China; 6 Institute of Human Nutrition, Columbia University, New York, New York, United States of America; 7 Departments of Biostatistics and Psychiatry, Columbia University, New York, New York, United States of America; Ecole Normale Supérieure de Lyon, France

## Abstract

This study aimed at substantiating the associations of the apolipoproein M gene (*APOM*) with type 2 diabetes (T2D) as well as with metabolic traits in Hong Kong Chinese. In addition, *APOM* gene function was further characterized to elucidate its activity in cholesterol metabolism. Seventeen *APOM* SNPs documented in the NCBI database were genotyped. Five SNPs were confirmed in our study cohort of 1234 T2D and 606 control participants. Three of the five SNPs rs707921(C+1871A), rs707922(G+1837T) and rs805264(G+203A) were in linkage disequilibrium (LD). We chose rs707922 to tag this LD region for down stream association analyses and characterized the function of this SNP at molecular level. No association between *APOM* and T2D susceptibility was detected in our Hong Kong Chinese cohort. Interestingly, the C allele of rs805297 was significantly associated with T2D duration of longer than 10 years (OR = 1.245, p = 0.015). The rs707922 TT genotype was significantly associated with elevated plasma total- and LDL- cholesterol levels (p = 0.006 and p = 0.009, respectively) in T2D patients. Molecular analyses of rs707922 lead to the discoveries of a novel transcript *APOM5* as well as the cryptic nature of exon 5 of the gene. Ectopic expression of *APOM5* transcript confirmed rs707922 allele-dependent activity of the transcript in modifying cholesterol homeostasis *in vitro*. In conclusion, the results here did not support *APOM* as a T2D susceptibility gene in Hong Kong Chinese. However, in T2D patients, a subset of *APOM* SNPs was associated with disease duration and metabolic traits. Further molecular analysis proved the functional activity of rs707922 in *APOM* expression and in regulation of cellular cholesterol content.

## Introduction

The human apolipoprotein M gene (*APOM,* Gene ID: 55937) is located on chromosome 6p21.33 and contains six exons spanning a region of 2.3 kb in length with gene structure conserved across species [1], [2]. In human and mice, *APOM* mRNA is highly expressed in liver and kidney [2]. The human apoM protein (MIM 606907) of 188 amino acids is mainly associated with HDL and to a minor degree with LDL, very low density lipoprotein, and chylomicrons [2]. Plasma apoM has been positively associated with plasma total cholesterol (TC), LDL cholesterol (LDL-C), and HDL cholesterol (HDL-C) [3]. *APOM* knockdown in mice by siRNA revealed its anti-atherosclerotic effect by participating in pre-β HDL formation and reverse cholesterol transport [4].

Kruit et al., recently reported the effect of cellular cholesterol accumulation on beta cell dysfunction in type 2 diabetes [5]. Such finding implies that factors (i.e., apoM) affecting the balance of cellular cholesterol content are likely to modify beta cell function and thus the susceptibility to or progression of type 2 diabetes.

Several additional lines of evidence also indicated the possible involvement of *APOM* in the development of diabetes and metabolic disturbances: 1) the human *APOM* gene is located within a high susceptibility region (6q21–q23) to type 2 diabetes (T2D) in genome-wide linkage analyses [6]. 2) SNP rs805296 (T-778C) in *APOM* promoter has been associated with the levels of plasma total cholesterol (TC) and fasting plasma glucose (FPG) in non-diabetic participants, 3) SNP rs805296 has also been associated with the susceptibility to T2D and coronary artery disease among the Northern Chinese [7], [8].

In 2010, China became the country with the largest diabetic population in the world. The Northern and Southern Chinese populations are distinct in genetic marker analyses [9], meaning disease markers identified in northen populations may not be shared by the Southern populations. The primary aim of the current study is to establish the association between *APOM* and T2D susceptibility in a Southern Chinese cohort in Hong Kong. By assuming the same effect size (OR = 1.934) and disease allele frequency as observed in the studies of Northern Chinese [8], the power of the current case-control study is over 95% with 1234 cases and 606 controls. The secondary aims are to examine for association between *APOM* and component metabolic traits as well as to further assess the function of the gene.

## Materials and Methods

### 1. Study population

The pilot cohort consisted of 103 male and 95 female controls (average age  =  43 yrs). They were Hong Kong Chinese adults recruited from a community health screening program of cardiovascular risk factors with normal response at a 75 g oral glucose tolerance test [10].

The study cohort had 1234 unrelated T2D patients and 606 controls. All participants gave written informed consent at the time of blood sampling. Ethics approval was obtained from the Clinical Research Ethics Committee of Chinese University of Hong Kong, Shatin, NT, Hong Kong. All T2D participants were selected from the Hong Kong Diabetes Registry. Control participants were recruited in a community health screening program for cardiovascular risk factors and some were hospital staff (3.1%, n = 19). No subdemographic differences were detected in control participants. All control participants had no known history of diabetes and had fasting plasma glucose (FPG) < 6.1 mmol/l.

Clinical assessments of participants had been described elsewhere [11]. Body mass index (BMI), blood pressure (BP) as well as fasting blood biochemical and metabolic profiles were measured. Among the 1234 T2D patients, 9.8% (n = 121) were on diet treatment only, 41.3% (n = 510) were on oral anti-diabetic drugs only, 12.5% (n = 154) were on insulin only, 9.5% (n = 117) on both oral anti-diabetic drugs and insulin, and 7.3% (n = 90) were treated for dyslipidemia.

### 2. Analysis and measurement

#### 2.1. SNP selection and genotyping analyses

Genomic DNA was prepared from whole blood as previously described [12]. Seventeen *APOM* SNPs including rs6921907, rs1266078, rs9267528, rs805297, rs4947251, rs9404941, rs805296, rs805264, rs3117581, rs34490746, rs11462733, rs2273612, rs707922, rs707921, rs28432254, rs3132449, rs3178094 enlisted in the NCBI database [13] were selected for genotyoping in the pilot cohort of 198 controls by multiplex reactions using the Mass ARRAY system (Sequenom, San Diego, CA, USA) at the Genome Quebec Innovation Centre, McGill University (Montréal, Quebec, Canada). Six of the seventeen SNPs were confirmed in the pilot cohort: rs1266078(T-1628G), rs805297(C-1065A), rs9404941(T-855C), rs805264(G+203A), rs707922(G+1837T) and rs707921(C+1871A). These six SNPs were further genotyped in the study cohort of 1840 participants (1234 cases and 606 controls). Case and control DNA samples were genotyped in parallel on the same plates. Two hundred ninety one duplicate samples (15.8%) were used to assess intra-plate and inter-plate genotype quality. No genotyping discrepancies were detected. The overall call rate was 98.0%. Five out of the six SNPs (except for rs1266078) were successfully genotyped in the study cohort.

#### 2.2. Plasma lipids and apoM levels

Plasma apoM concentration was estimated by dot-blot analysis using monoclonal mouse anti-human apoM antibody (ABNOVA, Taipei, Taiwan) following previously established protocols [14], [15]. Recombinant human apoM (ABNOVA, Taipei, Taiwan) was used as protein standard after serial dilution. The mean signal densities of each specimen and protein standards in triplicate measures were determined by ImageJ 1.42q software (http://rsbweb.nih.gov/ij/). ApoM concentration was derived from the standard curves developed using the recombinant apoM protein.

#### 2.3. Total cholesterol measures in cultured cells with ectopic *APOM1* and *APOM5* expression

WRL-68 and HepG2 hepatic cell lines were purchased from American Type Culture Collection (Rockville, MD, USA) and maintained in RPMI-1640 medium supplemented with 10% FBS and 100 units/ml penicillin and 100 mg/ml streptomycin in a humidified atmosphere containing 5% CO_2_ at 37°C. Prior to the expreiments measuring cellular and medium cholesterol content, cells were switched to serum free and phenol red free RPMI medium (Invitrogen, Carlsbad, CA, USA).

Ectopic expression of *APOM* was achieved by transient transfection of *APOM1* or *APOM5* cDNA (cloned into the pCMV-Myc vectors) into cultured cells at 70% confluence using LipofectamineTM 2000 reagent (Invitrogen, Carlsbad, CA, USA) following the manufactorer's protocols [16].

Cellular lipids were extracted as previously described [17]. Total cellular cholesterol was measured using the InfinityTM Cholesterol Liquid Stable Reagent (Thermo Fisher Scientific Inc., Middletown, VA, USA) following the manufacturer's instructions. The measured amount of total cholesterol was normalized by cell protein concentration.

### 3. Bioinformatics and molecular anayses of *APOM* transcripts

#### 3.1. Comparative genomic and protein sequence analyses

*APOM* transcript and gene sequences were obtained from the NCBI human Genome Browser [18], the Ensembl Genome Browser [19] and the human Expressed Sequence Tags (EST) databases. The Evolutionary Conserved Regions Browser [20] and the Ensembl Genome Browser were used to identify sequence conservation.

#### 3.2. Rapid amplification of cDNA ends (RACE)

The Human Liver FirstChoice RACE-ready cDNA kit (Ambion, Austin, Texas, USA) was used to amplify the 5′ and 3′ ends of novel *APOM* transcripts (Supplementary Figure S1, and Supplementary Table S1).

#### 3.3. Semi-quantitative RT–PCR analysis

Human normal adult tissue RNA samples were purchased commercially (Stratagene, La Jolla, CA, USA or Millipore Chemicon, Billerica, MA, USA). cDNA was synthesized using the GeneAmp RNA PCR kit (Applied Biosystems, Foster City, CA, USA) in combination with RNase inhibitor (Roche Applied Science, Indianapolis, IN, USA) and M-MuLV reverse transcriptase. The subsequent PCR amplification of cDNA was performed using the Ampli*Taq* Gold DNA Polymerase (Applied Biosystems, Foster City, CA, USA) following standardized protocols [21], [22]. The vulgate transcript *APOM1* (Ensembl: ENST00000375916) and the novel transcript *APOM5* were amplified using primer RT-PCR-YY12 paired with RT-PCR-YY13 and RT-PCR-YY12 paired with RT-PCR-YY14, respectively (Supplementary Table S4). GAPDH was amplified as a house-keeping gene control for RNA integrity and equal loading using the GAPDH primers, RT-PCR-Tao1 and RT-PCR-Tao2 [23] (Supplementary Table S1).

### 4. Statistics

Continuous variables were compared using Student's t test or one-way analysis of variance (ANOVA) for traits with normal distribution. Plasma triglycerides (TG) were skewed and logarithmically transformed. Association tests between genotypes and quantitative traits were performed in T2D patients and non-diabetic controls separately. Categorical variables, including genotype distributions were compared by χ^2^ tests.

Genotype distributions were tested for Hardy-Weinberg equilibrium using goodness-of-fit test (1 df). Informative missingness was checked by coding successful genotypes into one group and failed genotypes into another group followed by 2×2 Chi-square test for T2D and t-test for the quantitative traits of interest. One SNP (rs805264) with significant result (p<0.001) indicative of informative missingness (IM) was excluded for further T2D association analyses (Supplelmentary Table S2).

Pairwise LD of D' and r^2^ analyses were performed using Haploview (Broad Institute of MIT and Harvard, USA, version 4.0). 2×2 contingency tables were used for comparing the differences of allele frequencies and 2×3 contingency tables were used for detecting the differences of genotype frequencies between cases and controls. Allelic, dominant, recessive and additive genetic models were used to test the association between each SNPs and T2D. Multivariate logistic regression analysis was used to assess the significance of covariates and adjusted for confounders in the association of genetic factors with T2D.

The independent contributions of all traits, covariates, SNPs, and haplotypes were determined by multiple regression analysis. Association between haplotypes and T2D or metabolic traits were tested by Haploview (version 4.0, Broad Institute of MIT and Harvard, USA) and PHASE software (version 2.1, UW TechTransfer Digital Ventures, University of Washington, Seattle, WA, USA) [24]. When using PHASE software, the probability thresholds were set at 90% for haplotype inference to deal with ambiguous haplotypes. It was only used to infer the haplotype of each individual and thus the case-control permutation test was not conducted. The PHASE-imputed haplotypes were counted 20 times using different seed numbers. No difference between runs was detected.

To account for multiple testing, we used the Bonferroni correction and a statistical significance was considered only when an SNP association with T2D/metabolic traits was p<0.017 (equivelent to 0.05/3), and a haplotype association with T2D/metabolic traits was p<0.0125 (equivelent to 0.05/4). The human plasma apoM concentrations determined by dot-blot assays were compared by a nonparametric Kruskal-Wallis H test. A value of *p*<0.05 was considered significant.

Results from functional analyses were analyzed by Student's t-test for two-group comparison, and one way ANOVA for multiple group comparisons. A statistical significance is considered at p<0.05 level. All statistical analyses were performed using the SPSS program (SPSS version 15.0, Chicago, IL, USA) unless otherwise specified.

## Results

### 1. *APOM* genotype analyses

Seventeen *APOM* SNPs enlisted in the public databases were selected for genotyping in a pilot cohort of 198 control participants. Six SNPs were confirmed polymorphic in this pilot cohort of Hong Kong Chinese. These six SNPs were further genotyped in the full study cohort of 1840 participants. Five of the variants were successfully genotyped: rs805297(C-1065A), rs9404941(T-855C), rs805264(G+203A), rs707922(G+1837T), and rs707921(C+1871A) with genotype distributions fitting Hardy-Weinberg equilibrium. Table 1 summarized the allele and genotype frequencies of SNPs in non-diabetic controls and T2D patients. SNP rs805264 was removed from further association analysis for T2D due to informative missingness (Supplementary Table S2).

**Table 1:** *APOM SNP* genotype distributions, allele frequencies and association with T2D.

SNP ID	Group	Genotype n (% frequencies)			*p* value^a^	Allele n (% frequencies)		*p* value^b^	OR (95% CI)
C-1065A		CC	CA	AA		C	A		
rs805297	Control	273(46.6)	266(45.4)	47(8.0)	0.341	812(69.3)	360(30.7)	0.732	0.974(0.836–1.134)
	T2D	574(48.9)	493(41.9)	108(9.2)		1640(69.8)	709(30.2)		
T-855C		TT	CT	CC		T	C		
rs9404941	Control	345(57.1)	221(36.6)	38(6.3)	0.872	911(75.4)	297(24.6)	0.816	1.019(0.869–1.196)
	T2D	689(56.2)	464(37.8)	74(6.0)		1842(75.1)	612(24.9)		
G+203A		GG	GA	AA		G	A		
rs805264	Control	381(64.4)	178(30.1)	33(5.6)	0.188	940(79.4)	244(20.6)	0.404	0.929(0.782–1.104)
	T2D	798(64.9)	386(31.4)	46(3.7)		1982(80.6)	479(19.4)		
G+1837T		GG	GT	TT		G	T		
rs707922	Control	375(62.7)	188(31.4)	35(5.9)	0.180	938(78.4)	258(21.6)	0.201	0.895(0.755–1.061)
	T2D	786 (64.4)	386(31.6)	48(3.9)		1958(80.2)	483(19.8)		
C+1871A		CC	CA	AA		C	A		
rs707921	Control	385(64.1)	181(30.1)	35(5.8)	0.161	951(79.1)	251(20.9)	0.312	0.915(0.771–1.087)
	T2D	791(64.9)	380(31.2)	47(3.9)		1963(80.6)	475(19.4)		

a genotypic association;

b allelic association.

### 2. Association analysis

#### 2.1. *APOM* SNPs and T2D susceptibility

No significant association was detected between individual SNPs and T2D (Table 1). Further multiple logistic regression analysis adjusting for age, BMI, SBP (systolic blood pressure), DBP (diastolic blood pressure), TC and TG again detected no significant association between individual SNPs and T2D (data not shown).

#### 2.2. *APOM* SNPs and T2D duration

We next examine the association between *APOM* SNPs and T2D disease duration. T2D patients were subgrouped into disease duration of ≤ 10 years (n = 583) and disease duration of >10 years (n = 586). As shown in Table 2, the C allele of rs805297 was associated with T2D duration of longer than 10 years (odds ratio =  1.245, *p* = 0.015).

**Table 2:** Association between rs805297 (C-1065A) and T2D disease duration.

T2D duration (n)	Genotype n (%frequencies)			Allele n (% frequencies)		*p* value^a^	OR (95% CI)^b^
	CC	CA	AA	C	A	0.015	1.245(1.043–1.487)
≤10 years (583)	270(46.3)	249(42.7)	64(11.0)	789(67.7)	377(32.3)		
>10 years (586)	304(51.9)	239(40.8)	43(7.3)	847(72.3)	325(27.7)		

a allelic association;

b allelic odds ratio (OR) of association.

#### 2.3. *APOM* SNPs and metabolic traits

We next analyzed the association between SNPs and metabolic variables in patients and controls separately. SNP rs805297(C-1065A) was not associated with metabolic traits in either patients or controls. Since rs707922(G+1837T) was in near perfect LD with rs805264(G+203A) and rs707921(C+1871A) (Supplementary Figure S2B), similar association results were expected and observed. Table 3 showed the representative results using rs707922 as the marker SNP. Under recessive model, homozygous minor allele TT of rs707922 was associated with significantly higher TC (p = 0.006), LDL-C (p = 0.009) in T2D patients. In controls, no association between SNPs and metabolic traits was detected. When plasma apoM concentration was measured in T2D patients and controls subgrouped by their rs707922 genotype, the TT genotype was found associated with significantly higher apoM level as compared to the GT (and GG) genotype(s) (p = 0.002).

**Table 3:** rs707922 genotype and clinical characteristics of T2D patients and non-diabetic controls.

Phenotype	T2D							Non-diabetic control				
	Total T2D	TT	GT	GG	GT+GG	*p* value	Total control	TT	GT	GG	GT+GG	*p* value
n (Male/Female)	504/730	17/31	148/238	331/455	479/693	0.460	275/331	19/16	77/111	176/199	253/310	0.281
AGE (yrs)	50.03±13.71	52.08±13.07	51.17±13.81	49.53±13.63	50.07±13.69	0.316	41.45±10.44	43.94±7.13	41.21±10.77	41.29±10.51	41.27±10.59	0.141
DM duration (yrs)	9.21±6.91	10.35±1.30	9.55±0.36	9.05±0.24	9.22±0.20	0.574	-	-	-	-	-	-
BMI (kg/m^2^)	25.26±4.20	24.76±3.88	25.26±4.18	25.27±4.22	25.27±4.21	0.544	22.93±3.28	23.42±2.74	23.21±3.45	22.69±3.21	22.86±3.30	0.603
SBP (mmHg)	134.70±22.94	137.09±26.79	133.74±23.42	135.16±22.52	134.74±22.81	0.625	115.37±16.42	115.58±16.08	116.25±17.06	114.70±16.17	115.23±16.48	0.402
DBP (mmHg)	76.93±11.22	75.96±12.08	76.09±11.48	77.34±11.05	76.96±11.20	0.711	72.30±11.26	75.32±10.41	72.78±11.91	71.74±11.04	72.09±11.34	0.361
HbA1_c_ (%)	7.93±1.85	8.65±2.44	7.88±1.81	7.90±1.82	7.90±1.81	0.031	-	-	-	-	-	-
FPG (mmol/l)	8.95±3.51	10.17±3.87	8.77±3.68	8.95±3.38	8.90±3.48	0.022	4.84±0.41	4.95±0.34	4.84±0.43	4.83±0.41	4.84±0.42	0.246
TC (mmol/l)	5.45±1.25	6.05±1.41	5.31±1.15	5.48±1.28	5.43±1.24	0.006*	5.04±0.95	5.17±0.80	4.93±1.03	5.08±0.92	5.03±0.96	0.759
HDL-C (mmol/l)	1.27±0.37	1.37±0.47	1.26±0.35	1.26±0.37	1.26±0.36	0.115	1.56±0.42	1.54±0.39	1.56±0.43	1.56±0.42	1.56±0.43	0.757
TG (mmol/l)	1.42 (0.73–2.76)	1.46 (0.74–2.88)	1.37 (0.71–2.65)	1.43 (0.73–2.81)	1.41 (0.73–2.76)	0.918	0.93 (0.53–1.61)	1.11 (0.60–2.02)	0.90 (0.52–1.55)	0.92 (0.53-1.60)	0.92 (0.53–1.58)	0.180
LDL-C (mmol/l)	3.39±1.00	3.85±1.20	3.31±0.91	3.40±1.03	3.37±0.99	0.009*	2.99±0.83	3.03±0.73	2.94±0.87	3.02±0.81	2.99±0.83	0.763
apoM (µg/µl)^+^	0.049±0.02	0.06±0.007	0.046±0.022	0.036±0.021	0.042±0.022	0.002*	0.056±0.02	0.06±0.018	0.06±0.011	0.04±0.018	0.052±0.02	0.059

Values are number of subjects, mean ± SD, or geometric mean (95% confidence interval). *P* values represent the comparison between subgroup with TT genotype and subgroup with TG or GG genotype (a recessive model), *P* values are adjusted for age, sex, BMI, disease duration, SBP, DBP, HbA1_c_, FPG, TC, HDL-C, TG, LDL-C in T2D. In non-diabetic controls, the *p* values are adjusted for age, sex and BMI. ^+^Plasma apoM concentration was measured in part of the study cohort: 62 cases (n of genotypes TT/GT/GG  =  16/23/23) and 68 controls (n of genotypes TT/GT/GG  =  22/23/23).

* Statistical significance (*P*<0.017).

### 3. *APOM* haplotype association with T2D and metabolic profiles

As mentioned above, rs805264, rs707922, and rs707921 are located within the same LD block. Therefore, in the subsequent haplotype analysis, rs707922 was used to ‘tag’ the three SNPs. Haplotype construction was conducted for rs707922(G+1837T) with other independent SNPs rs805297(C-1065A) and rs9404941(T-855C). Four haplotypes (A-T-G, C-C-G, C-T-G, and C-T-T) accounting for 99.9% of all possible haplotypes were detected in our Hong Kong Chinese population (Supplementary Table S3). No significant association was found between these haplotypes with T2D. Homozygous C-T-T was significantly associatiated with elevated TC, LDL-C and HbA1c in T2D patients (*p*<0.0125, Supplementary Table S4).

### 4. Bioinformatics and molecular analyses of *APOM* transcripts

#### 4.1. Bioinformatics analyses of *APOM* transcripts

**Figure 1:**
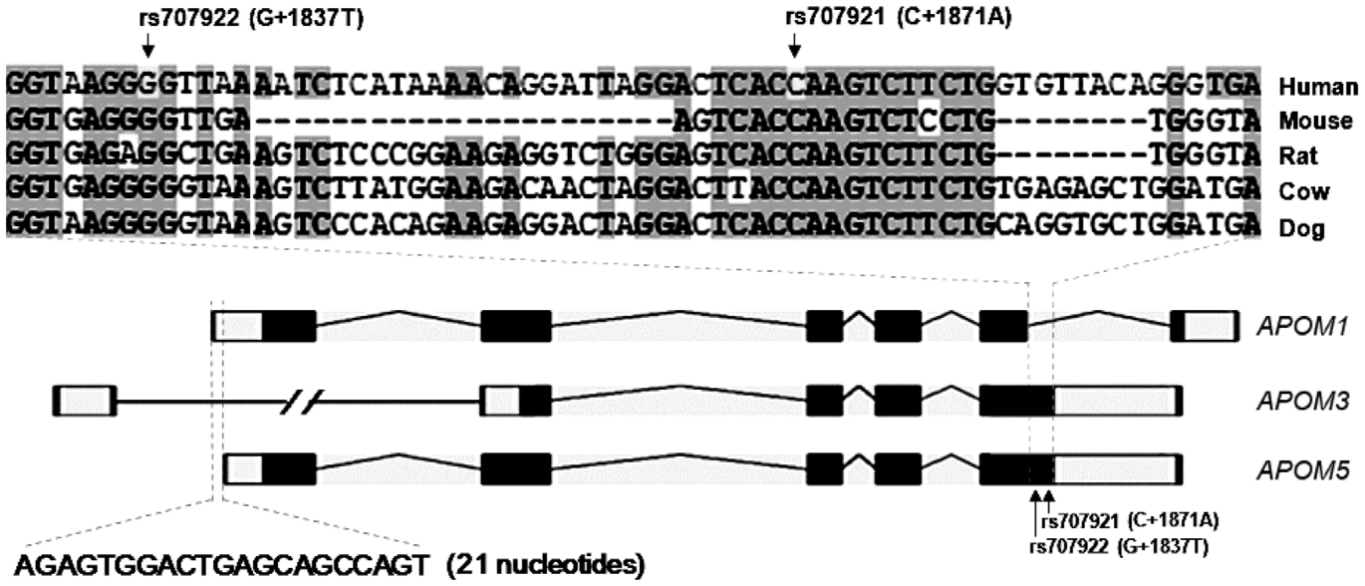
Cross-species sequence alignment and information of *APOM* transcripts. Transcript patterns were constructed by Ensembl browser tool. Top panel (sequence view): Fragments spanning SNPs rs707922(G+1837T) and rs707921(C+1871A) were aligned among different species including human, mouse, rat, cow and dog. Both SNPs, as indicated by arrowheads, fell within the highly conserved region (shaded in grey). Bottom panel (structure view): A schematic representation of the structure of human *APOM5* transcript compared with those of the *APOM1* and *APOM3* with the positions of coding exons (black boxes), introns (horizontal lines) and untranslated exons (white boxes) indicated. Arrowheads followed by vertical dashed lines indicated the corresponding positions of rs707922 and rs707921 on different *APOM* transcripts. Also indicated by the dashed lines at the 5′ end of the gene structures was the 21-nucleotide sequence difference between *APOM1 a*nd *APOM5* transcripts.

Sequence alignment analysis revealed conserved regions of the *APOM* gene in human, mouse, rat, cow, and dog (Supplementary Figure S3). SNPs rs707922 (G+1837T) and rs707921 (C+1871A) which associated with plasma levels of TC, LDL-C and apoM in T2D patients fell within the evolutionary conserved region. Figure 1 (top panel) illustrated the cross-species sequence conservation of the rs707922- and rs707921- flanking region. BLAST search using this conserved sequence as the template returned unique human EST clones BI757556 (human brain) and AA975560.1 (human kidney) which are likely other *APOM* transcripts. The Ensembl Browser also displayed three *APOM* transcripts (*APOM1:* Ensembl-ENST00000375916, *APOM2*: Ensembl-ENST00000375920 and *APOM3:* Ensembl-ENST00000375918).

#### 4.2. Molecular cloning of *APOM* transcripts

Since the sequences of EST clones BI757556 and AA975560.1 were different than the known *APOM* transcripts, we proceeded with cloning alternative transcripts of *APOM*. A novel transcript, designated *APOM5*, was identified by 5′ RACE and 3′ RACE. As shown in Figure 1, the 3′ end of *APOM5* was identical to the 3′ end of *APOM3*. The 5′ end, however, was similar to that of the vulgate *APOM* transcript (designated *APOM1*) except that the transcription start site of *APOM5* was 21 nucleotides downstream that of the *APOM1*. The full-length sequence of *APOM5* is provided in Supplementary Figure S4. It is important to note that the two SNPs rs707922(G+1837T) and rs707921(C+1871A) associated with metabolic traits in T2D are located to the exon 5 of *APOM5*. On the contrary, when reference to the vulgate *APOM1* transcript, rs707921 and rs707922 are located to intron 5. These observations support the cryptic nature of exon 5 of the gene.

#### 4.3. Tissue expression and function of *APOM* transcripts

**Figure 2:**
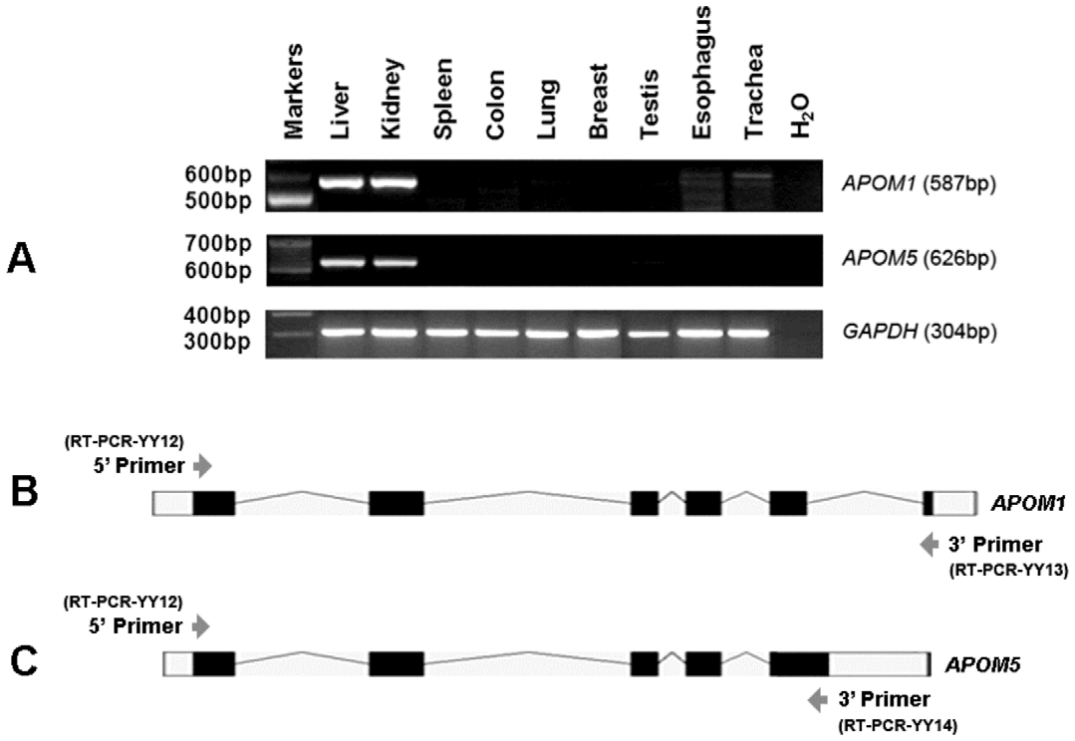
Tissue expression of human *APOM1* and *APOM5*. (A) Commercial human tissue RNA panels were used for cDNA synthesis by the GeneAmp RNA PCR kit. Primers specific to *APOM* transcripts or GAPDH (details provided in Materials and Methods) were used for cDNA amplfication. The molecular sizes of DNA markers and PCR products are indicated on the left and right side of the panel, respectively. Primer positions are indicated by arrowheads in the schematic representations of *APOM*1 transcript (B), and *APOM*5 transcript (C). Positions of coding exons (black boxes), introns (fold lines), untranslated region (white boxes) are indicated.

Tissue expression of *APOM5* transcript was determined by RT-PCR. As shown in Figure 2, *APOM5* and the vulgate transcript *APOM1* shared similar tissue expression profiles: relatively strong expression in liver and kidney and almost undetectable in spleen, colon, lung, breast, testis, esophagus, and trachea. Figrue 3 showed that the expression of two allelic forms of *APOM5* transcripts elicited distinct effects on cellular cholesterol content relative to *APOM1*. Expression of the transcript in G-allelic form (*APOM5*-G) resulted in slight accumulation of cholesterol in the cells whereas cells expressing the T-allelic form (*APOM5*-T) of the transcript had lower cholesterol content. Consistent results were obtained in two human hepatic cell lines HepG2 and WRL68.

**Figure 3:**
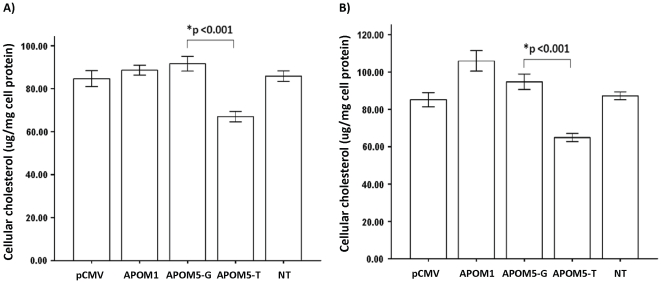
*ApoM5* transcript affects cellular cholesterol content in an SNP-dependent manner. Vector control (pCMV), *APOM1-*, as well as *APOM5-* encoding constructs were transfected into hepatic cells maintained in serum-free medium 24 hours prior to lipid extraction and cholesterol measurement. (A). HepG2 cells. (B). WRL68 cells. *APOM1* represents the vulgate *APOM* transcript. *APOM5* construct encoding the G or T allelic forms of the transcript at the position corresponding to SNP rs707922 are designated *APOM5-G* or *APOM5-T*, respectively. NT: no transfection. Total cholesterol concentrastion in cells was normalized by the total amount of cellular protein. Results represented the mean ± S.E.M of experiments in triplicates. * Significant difference between G and T allele-expressing cells (p<0.05).

## Discussion

The results of this study did not support an assoiation between *APOM* and T2D suseptibility in Hong Kong Chinese. For a subset of SNPs, we presented evidence of association between *APOM* and disease duration as well as metabolic traits in T2D patients. Further characterization of rs707922, one of the metabolic trait-associated SNP at molecular level lead to the discoveries of a novel transcript *APOM5* and its SNP-dependent effect on cellular cholesterol content.

The LD block formed among rs805264, rs707922, and rs707921 in our cohort agreed with the LD structure reported in the Northern Chinese [25]. It is currently unknown whether this subset of SNPs is also associated with metabolic traits in Northern Chinese with T2D. Among the four common haplotypes constructed from rs805297, rs9404941, and rs707922, only homozygous haplotype C-T-T was significantly associated with higher TC, LDL-C and HbA1c levels in T2D patients. Given the established association between rs7070922 and plasma TC and LDL-C levels, these association results did not support additional effects of the haplotypes on serum cholesterol levels. Interestingly, the association between the homozygous haplotype C-T-T with HbA1c indicated the interaction among the three alleles to control systemic glucose level in T2D patients.

It would have been ideal if the previously reported association between SNP rs805296(T-778C) and T2D in Northern Chinese were reproduced in this Hong Kong Chinese population. Unfortunately, genotyping of this SNP failed to produce results in this study, precluding it being used for discussions attempting to reconcile the current findings with prior results. Although rs805296 is physically close to rs9404941, the existing information/data does not allow the relationship between SNP rs805296 and T2D/T2D metabolic traits to be predicted in our cohort.

It is noteworthy that the case-control study design adopted by the current and other studies tend to be limited by the heterogeneity of the prevalent cases with regards to T2D ascertainment, i.e., both those have developed T2D and those have survived in the setting of T2D were included as cases. Therefore, those ‘susceptible to’ the disease were not distinguished from those ‘survived’ the disease'. One possibility to circumvent such issue is to examine for similar duration of diabetes across studies being compared and test for difference in duration of T2D by SNP. Interestingly, while our results did not support an association between *APOM* and T2D susceptibility, stratification of our cases by disease duration allowed us to detect an association between rs805297(C-1065A) and T2D duration. This result implied the possibility that relative to the rs805297-A carriers, the rs805297-C carriers better survived the diabetic condition over the long term and such possibility can be further tested. Interestingly, Zhao et al., recently reported a positive association between rs805297-A and the risk of stroke in Norhtern Chinese (OR = 1.38, p  = 0.002) after adjusting for other risk factors including history of diabetes [26]. Whether such association is present among the Southern Chinese requires further investigation. Nevertheless, losing rs805297-A carriers with T2D to stroke over time provides a plausible explanation for the observed higher frequency of rs805297-C allele in the T2D duration >10 years subgroup (relative to T2D duration ≤10 years subgroup).

Previous studies attempting to correlate plasma apoM and cholesterol levels have generated inconsistent results [3], [27]. In this study, rs707922 homozygous minor allele (TT) was associated with elevated TC and LDL-C as well as plasma apoM levels in diabetic cases (average BMI of 25.26). These observations are consistent with previously reported positive association between plasma apoM and plasma TC and LDL-C in overweight-obese individuals [3]. Results presented by Han et al. from the study of a Northern Chinese cohort showed significant association between rs707922 T allele and increased risk of cerebral infraction (OR = 1.78, p = 0.000). In parallel they also confirmed hypercholesterolemia as an independent risk factor for cerebral infraction [25]. These results implied the possibility that rs707922 is also a modifier of serum cholesterol in Northern Chinese. The association between rs707922 TT genotype and elevated serum total-/LDL- cholesterol levels in type 2 diabetes found in the current report deserves to be further substantiated in strict replicate studies.

The mechanism underlying the effects of rs707922 on plasma TC and LDL-C levels in diabetes remains elusive. Richter et al., reported HNF-1 alpha being a potent transcription activator of *APOM* [28]. The decreased serum apoM level in maturity-onset diabetes of the young subjects as compared to the controls could be explained by the HNF-1 alpha mutations in these patients [28]. Given the association between *APOM* and metabolic traits found in this study, one may speculate that SNP rs707922 (G+1837T), in the capacity of an intronic SNP (reference to the *APOM1* transcript), may modify *APOM* expression through SNP-specific recruitment of transcription factors (i.e., PAX 6 showed an allele-specific interaction with rs707922 T by computer prediction as presented in Supplementary Figure S5) and subsequently affect cellular cholesterol homeostasis in liver and possibly other tissues. More interestingly, we found that rs707922 can also assume the capacity as an exonic SNP (i.e., reference to the *APOM5* transcript). While the function of *APOM5* requires further elucidation, the high renal and hepatic expression levels of *APOM1* and *APOM5* indicated the possibility of these transcripts coordinate to regulate cholesterol homeostasis in these tissues. Such possibility is further supported by the results showing the activities of ectopically expressed *APOM5* in modifying hepatic cell cholesterol content. With regards to systematic cholesterol homeostasis, we observed that homozygous rs707922-T allele associated with elevated total- and LDL-cholesterol levels. One possible mechanism of such elevation is through reducd hepatic and/or pheripheral clearance of circulating cholesterol. Consistent with this notion, our *in vitro* data showed that hepatic cells over-expressing *APOM5-T* transcript had lower cholesterol content relative to cells expressing the *APOM5-G* counterpart.

In conclusion, the *APOM* SNP frequencies and the LD structure reported in this study of Hong Kong Chinese population will facilitate future population genetics studies. While our results did not support an association between *APOM* and T2D susceptibility in Hong Kong Chinese, subgroup analyses found SNP as well as haplotype associations between *APOM* and metabolic traits in T2D. Bioinformatics/molecular analyses revealed the cryptic nature of exon 5 responsible for the expression of a novel transcript *APOM5*, predominantly in liver and kidney. The activity of *APOM5* on modifying cellular cholesterol content revealed another layer of regulation underlying the expression and function of *APOM*.

## Supporting Information

Figure S1 **Positions of primers used for 5′ and 3′ RACE experiments.** The relative positions of primers were indicated by pink arrowheads above or below the *APOM*5 transcripts with the positions of coding exons (dark blue boxes), introns (fold lines), untranslated exons (open boxes) and adaptors (red boxes) indicated. The primer names corresponding to those in the Supplemental Table S1 were indicated in parentheses. (**A**) Positions of the primers used for 5′ RACE experiment. (**B**) Positions of the primers used for 3′ RACE experiment. (**C**) A schematic representation of the structure of *APOM1* transcript in the same length proportion to the *APOM5* transcript. Since all 3′ primers were targeted to *APOM5*-specific sequences, no amplification *APOM1* transcript was expected.
(TIFF)

Figure S2 **Linkage disequilibrium (LD) structure of five SNPs in the full cohort (n = 1840).** Images were taken from HaploView 4.0. Blocks were defined using the solid spline of linkage disequilibrium. A. The red squares without numbers indicates the D' value of 1.00. B. The shades of grey refer to the strength of pairwise linkage disequilibrium based on r^2^, which was also indicated within each square. White squares were very low value of r^2^. Black squares indicate r^2^ close to or equal to 1. The pilot cohort produced very similar LD structure results.
(TIFF)

Figure S3 **Conservation profiles and transcript patterns of the** ***APOM*** **.** The conservation profiles (percent identity cut-off of 50% to 100%) of the human *APOM1* (shown on the very top of the figure) in comparison with the mouse (*Mus musculus*; chr17), rat (*Rattus norvegicus;* chr20), cow (*Bos Taurus;* chr23) and dog (*Canis familiaris;* chr12) genes are shown. Conserved sequences were defined as coding exons (blue), The Evolutionary Conserved Regions (ECRs) were indicated by pink lines (on top of the panel for each species) with a default value of 70%. The human *APOM* was depicted as a horizontal blue line above the graph, with strand/transcriptional orientation indicated by arrows. *APOM* coding exons were shown as blue boxes along the line, while untranslated regions (UTR) were indicated as yellow boxes. Peaks within the conservation profile which corresponded to these five exons of *APOM* were similarly coloured within the plot. Peaks within the conservation profile that did not correspond to transcribed sequences were highlighted in red colour. Regions of transposable elements and simple repeats were highlighted in green color. Relative length was indicated by a line at the bottom for human *APOM* (chr6: 31730492-31733971). The locations of *APOM* SNPs, rs805297 (C-1065A), rs9404941 (T-855C), rs805264 (G+203A), rs707922 (G+1837T) and rs707921 (C+1871A) were indicated by arrowheads on the top of the figure.
(TIFF)

Figure S4 **Sequencing result of** ***APOM5*** **transcript from 5′-RACE.** Shown above is the reverse complementary sequence with the location corresponding to the first nucleotide of the cDNA sequence listed in Supplementary Figure S3 indicated by black arrowhead. The nucleotides between the black arrowhead and the red arrowhead are identical to the sequence shown in Supplementary Figure S3 (from nucleotide position 1 to 655). The forward sequence corresponding to the above chromatograph is provided below for easy viewing. ↓ 5′AGGGGAGAGAGCAGTTAAGGCACACAGAGCACCAGCTCCCTCCTGCCTGAAGATGTTCCACCAAATTTGGGCAGCTCTGCTCTACTTCTATGGTATTATCCTTAACTCCATCTACCAGTGCCCTGAGCACAGTCAACTGACAACTCTGGGCGTGGATGGGAAGGAGTTCCCAGAGGTCCACTTGGGCCAGTGGTACTTTATCGCAGGGGCAGCTCCCACCAAGGAGGAGTTGGCAACTTTTGACCCTGTGGACAACATTGTCTTCAATATGGCTGCTGGCTCTGCCCCGATGCAGCTCCACCTTCGTGCTACCATCCGCATGAAAGATGGGCTCTGTGTGCCCCGGAAATGGATCTACCACCTGACTGAAGGGAGCACAGATCTCAGAACTGAAGGCCGCCCTGACATGAAGACTGAGCTCTTTTCCAGCTCATGCCCAGGTGGAATCATGCTGAATGAGACAGGCCAGGGTTACCAGCGCTTTCTCCTCTACAATCGCTCACCACATCCTCCCGAAAAGTGTGTGGAGGAATTCAAGTCCCTGACTTCCTGCCTGGACTCCAAAGCCTTCTTATTGACTCCTAGGAATCAAGGTAAGGGGTTAAAATCTCATAAAACAGGATTAGGACTCACCAAGTCTTCTGGTGTTACAGGG-3′ ↑
(TIFF)

Figure S5 **Computer-predicted transcription factor interaction sites in nucleotide sequences spanning SNPs rs707922(G+1837T) and rs707921(C+1871A).** This figure is generated by the MATCH program. **Top panel:** The transcription factors predicted to interact with the nucleotide sequences spanning the major allele of SNPs rs707922 (G allele) and rs707921 (the C allele). **Bottom panel:** The transcription factors predicted to interact with the nucleotide sequences spanning the minor allele of SNPs rs707922 (the T allele) and rs707921 (the A allele). The predicted transcription factors are marked by blue text with scores of matrix match indicated in parentheses. The locations and orientations of the binding sites for these predicted transcription factors are marked by black horizontal dashed lines with arrows. Highlighted in pink boxes are allele-specific transcription factors (PAX6 and AREB6 for rs707922-T; HNF4 and OCT1 for rs707921-A) and their corresponding binding sites. The vertical dashed lines indicate the locations of SNPs rs707922 and rs707921. The precise nucleotide positions of SNP rs707922 and rs707921 are also highlighted with grey boxes in the DNA sequences represented by red colored text.
(TIFF)

Table S1 **Sequences of primers used in this study.**
(PDF)

Table S2 **Summary of data quality of the five successfully genotyped** ***APOM*** **SNPs in the full cohort (n = 1840).** The clinical traits tested for IM include T2D, TG, HbA_1c_, FPG, TC, HDL-C, and LDL-C.
(PDF)

Table S3 **Frequencies of common haplotypes constructed by APOM SNPs rs805297, rs904941, and rs707922.**
(PDF)

Table S4 **Haplotype C-T-T formed from SNPs rs805297(C-1065A), rs9404941(T-855C), and rs707922 (G+1837T) and clinical characteristics of T2D patients.** Values are either number of subjects, mean ± SD, or geometric mean (95% confidence interval). *p* values here represent the comparisons between subgroup of homozygotes of C-T-T haplotype vs. subgroup with one C-T-T haplotype and without C-T-T haplotype (a recessive model). *p* values are adjusted for age, sex, BMI and disease duration in T2D. In non-diabetic controls, the *p* values without parentheses are adjusted for age, sex and BMI. “+/+” represent the homozygote of haplotype C-T-T, “+/−” represent the heterozygote of haplotype C-T-T, “−/−” represent the subgroup who do not have the haplotype of C-T-T. Individuals on lipid lowering medications (n = 90) were excluded for association analysis with lipid traits. ^*^ statistical significance (p<0.0125).
(PDF)
