# Supplementary figures and images for: Apolipoprotein M Gene (APOM) Polymorphism Modifies Metabolic and Disease Traits in Type 2 Diabetes
Source: PLoS One. 2011 Feb 24;6(2):e17324. doi: 10.1371/journal.pone.0017324 (PMC3044746; doi:10.1371/journal.pone.0017324)

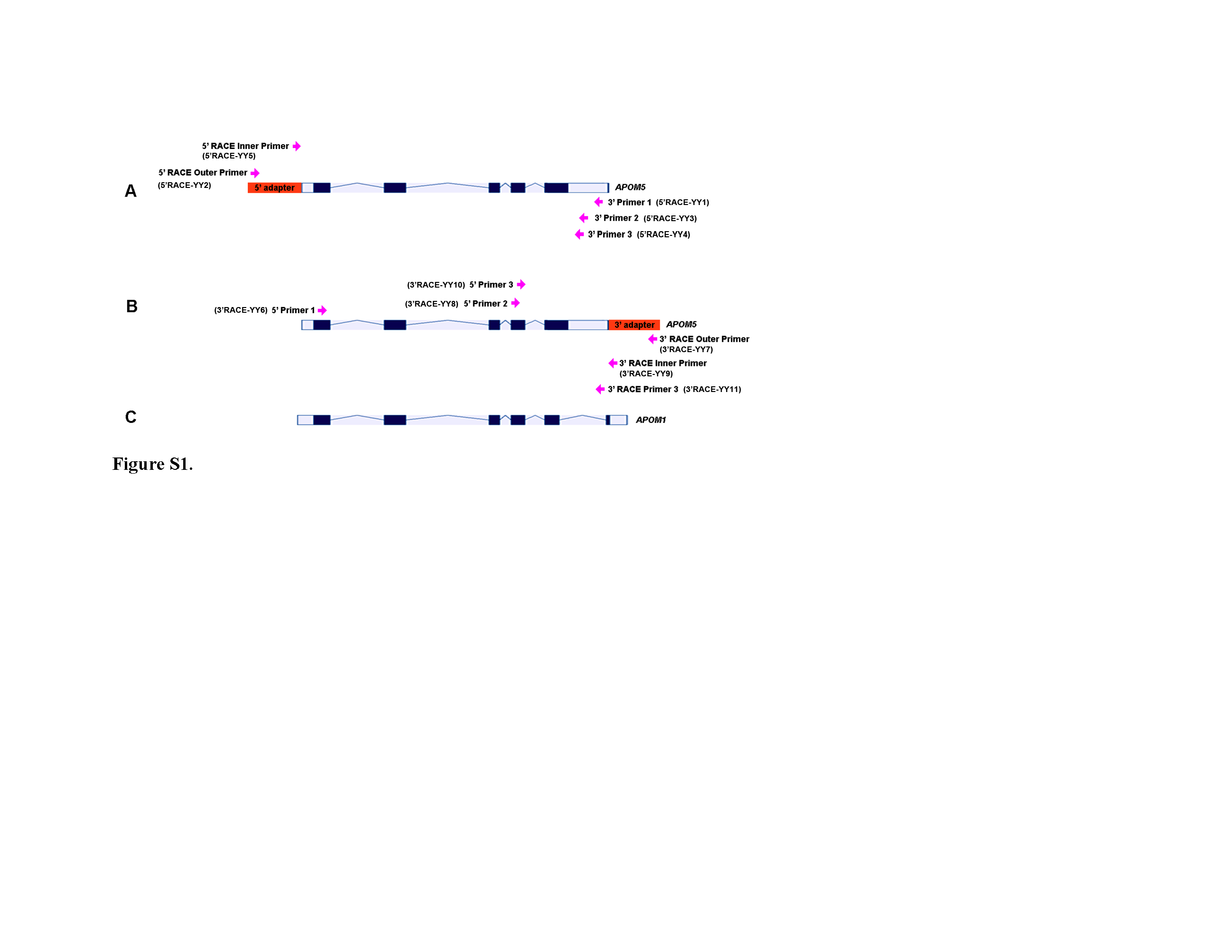

Supplement: Figure S1 — Positions of primers used for 5′ and 3′ RACE experiments. The relative positions of primers were indicated by pink arrowheads above or below the APOM5 transcripts with the positions of coding exons (dark blue boxes), introns (fold lines), untranslated exons (open boxes) and adaptors (red boxes) indicated. The primer names corresponding to those in the Supplemental Table S1 were indicated in parentheses. (A) Positions of the primers used for 5′ RACE experiment. (B) Positions of the primers used for 3′ RACE experiment. (C) A schematic representation of the structure of APOM1 transcript in the same length proportion to the APOM5 transcript. Since all 3′ primers were targeted to APOM5-specific sequences, no amplification APOM1 transcript was expected. (TIFF) [file pone.0017324.s001.tiff]

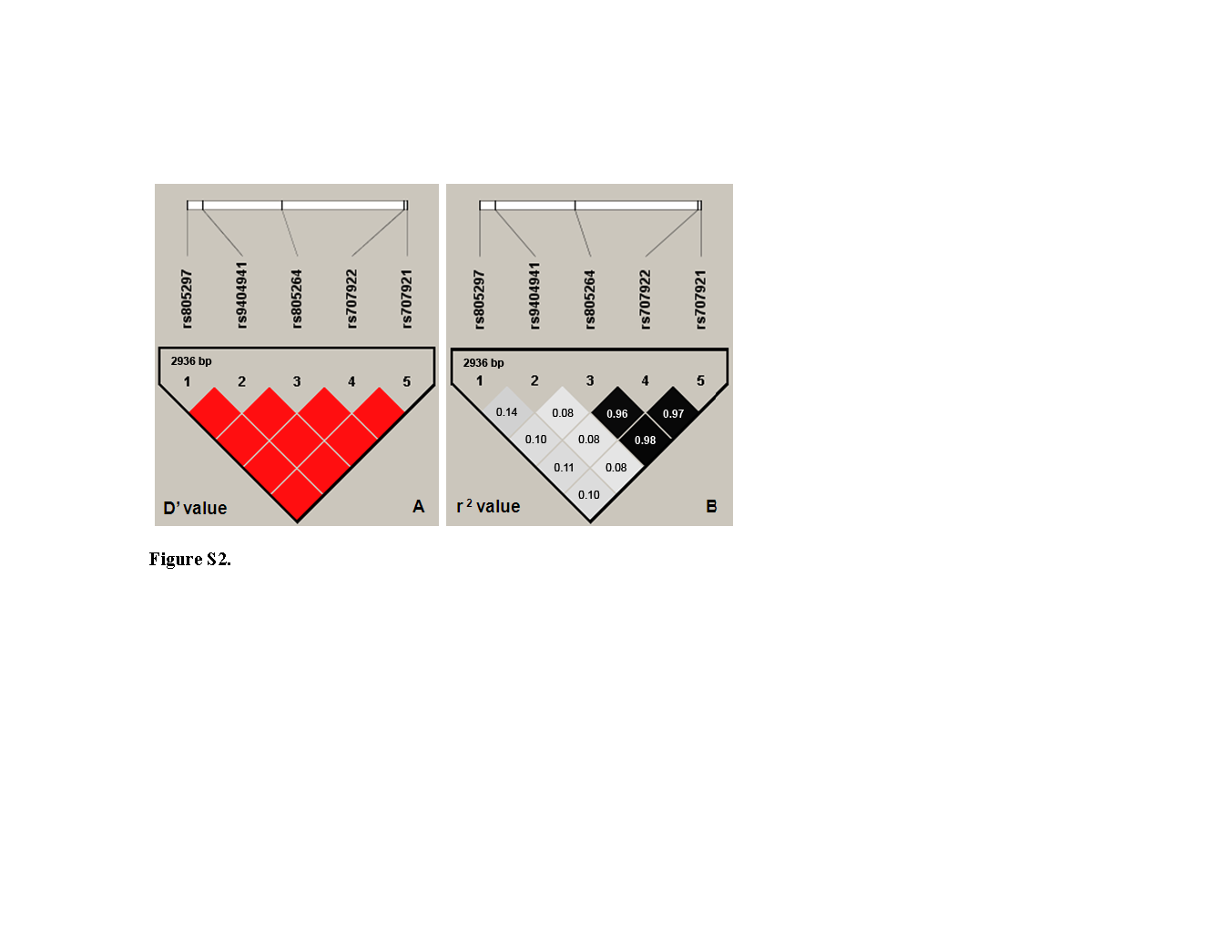

Supplement: Figure S2 — Linkage disequilibrium (LD) structure of five SNPs in the full cohort (n = 1840). Images were taken from HaploView 4.0. Blocks were defined using the solid spline of linkage disequilibrium. A. The red squares without numbers indicates the D' value of 1.00. B. The shades of grey refer to the strength of pairwise linkage disequilibrium based on r2, which was also indicated within each square. White squares were very low value of r2. Black squares indicate r2 close to or equal to 1. The pilot cohort produced very similar LD structure results. (TIFF) [file pone.0017324.s002.tiff]

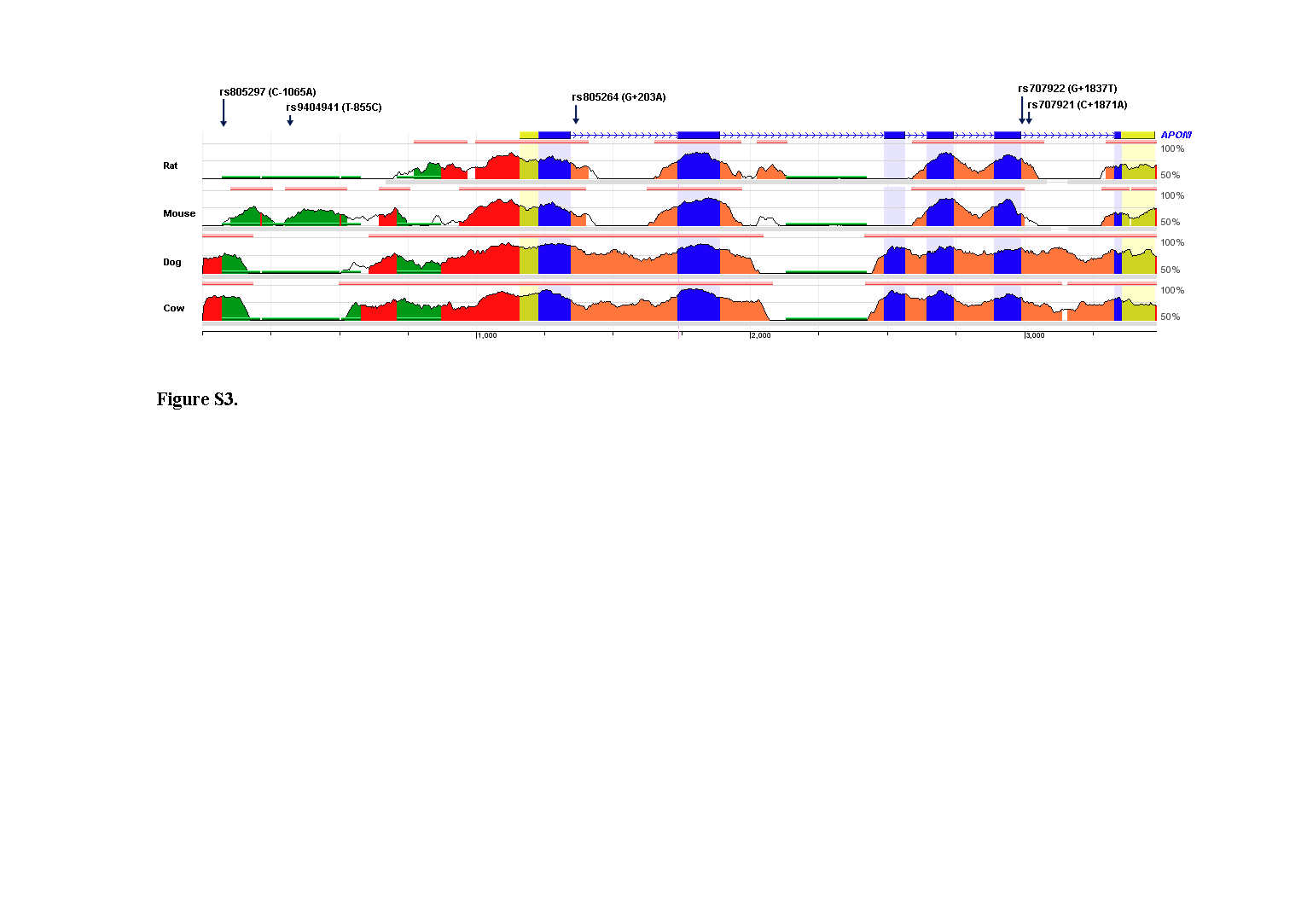

Supplement: Figure S3 — Conservation profiles and transcript patterns of the APOM . The conservation profiles (percent identity cut-off of 50% to 100%) of the human APOM1 (shown on the very top of the figure) in comparison with the mouse (Mus musculus; chr17), rat (Rattus norvegicus; chr20), cow (Bos Taurus; chr23) and dog (Canis familiaris; chr12) genes are shown. Conserved sequences were defined as coding exons (blue), The Evolutionary Conserved Regions (ECRs) were indicated by pink lines (on top of the panel for each species) with a default value of 70%. The human APOM was depicted as a horizontal blue line above the graph, with strand/transcriptional orientation indicated by arrows. APOM coding exons were shown as blue boxes along the line, while untranslated regions (UTR) were indicated as yellow boxes. Peaks within the conservation profile which corresponded to these five exons of APOM were similarly coloured within the plot. Peaks within the conservation profile that did not correspond to transcribed sequences were highlighted in red colour. Regions of transposable elements and simple repeats were highlighted in green color. Relative length was indicated by a line at the bottom for human APOM (chr6: 31730492-31733971). The locations of APOM SNPs, rs805297 (C-1065A), rs9404941 (T-855C), rs805264 (G+203A), rs707922 (G+1837T) and rs707921 (C+1871A) were indicated by arrowheads on the top of the figure. (TIFF) [file pone.0017324.s003.tiff]

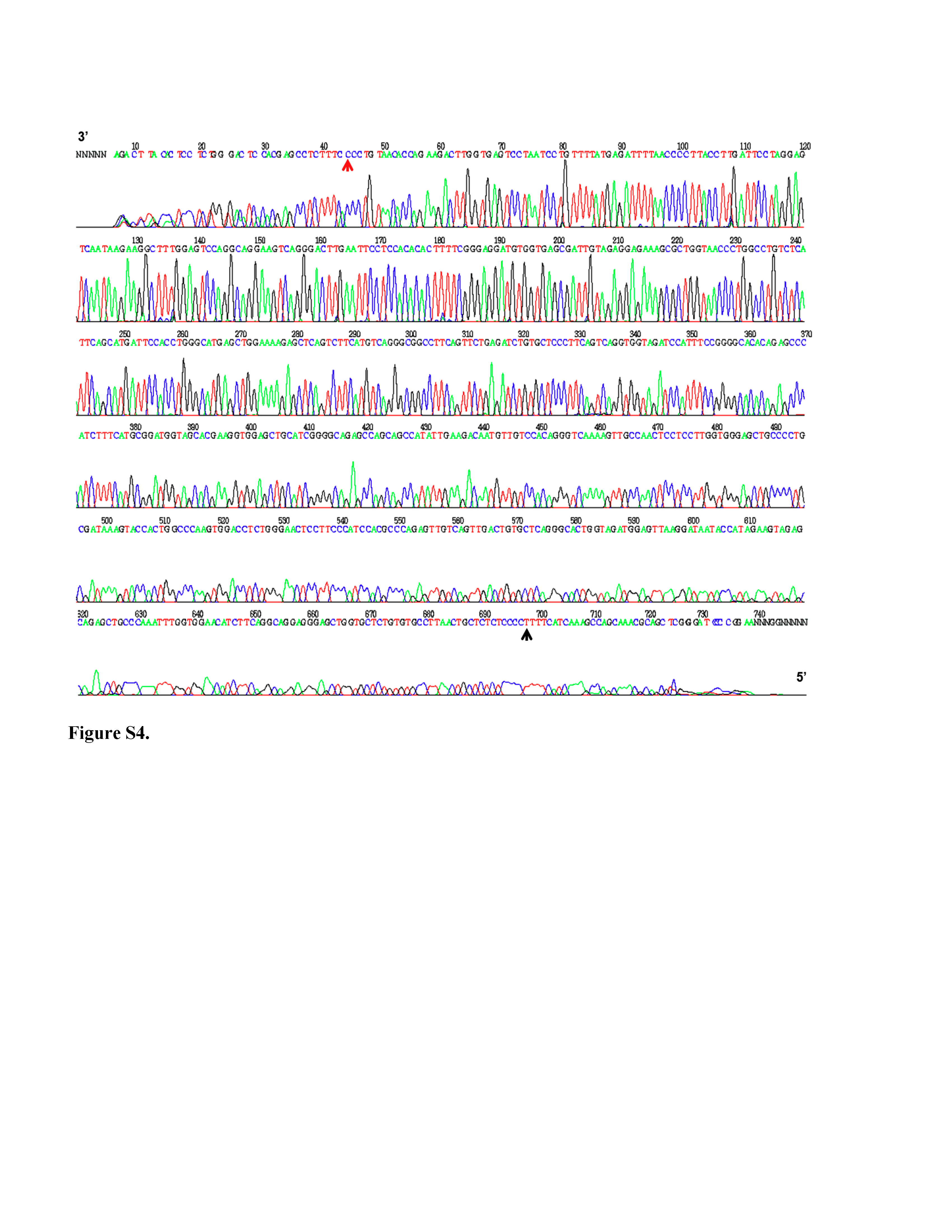

Supplement: Figure S4 — Sequencing result of APOM5 transcript from 5′-RACE. Shown above is the reverse complementary sequence with the location corresponding to the first nucleotide of the cDNA sequence listed in Supplementary Figure S3 indicated by black arrowhead. The nucleotides between the black arrowhead and the red arrowhead are identical to the sequence shown in Supplementary Figure S3 (from nucleotide position 1 to 655). The forward sequence corresponding to the above chromatograph is provided below for easy viewing. ↓ 5′AGGGGAGAGAGCAGTTAAGGCACACAGAGCACCAGCTCCCTCCTGCCTGAAGATGTTCCACCAAATTTGGGCAGCTCTGCTCTACTTCTATGGTATTATCCTTAACTCCATCTACCAGTGCCCTGAGCACAGTCAACTGACAACTCTGGGCGTGGATGGGAAGGAGTTCCCAGAGGTCCACTTGGGCCAGTGGTACTTTATCGCAGGGGCAGCTCCCACCAAGGAGGAGTTGGCAACTTTTGACCCTGTGGACAACATTGTCTTCAATATGGCTGCTGGCTCTGCCCCGATGCAGCTCCACCTTCGTGCTACCATCCGCATGAAAGATGGGCTCTGTGTGCCCCGGAAATGGATCTACCACCTGACTGAAGGGAGCACAGATCTCAGAACTGAAGGCCGCCCTGACATGAAGACTGAGCTCTTTTCCAGCTCATGCCCAGGTGGAATCATGCTGAATGAGACAGGCCAGGGTTACCAGCGCTTTCTCCTCTACAATCGCTCACCACATCCTCCCGAAAAGTGTGTGGAGGAATTCAAGTCCCTGACTTCCTGCCTGGACTCCAAAGCCTTCTTATTGACTCCTAGGAATCAAGGTAAGGGGTTAAAATCTCATAAAACAGGATTAGGACTCACCAAGTCTTCTGGTGTTACAGGG-3′ ↑ (TIFF) [file pone.0017324.s004.tiff]

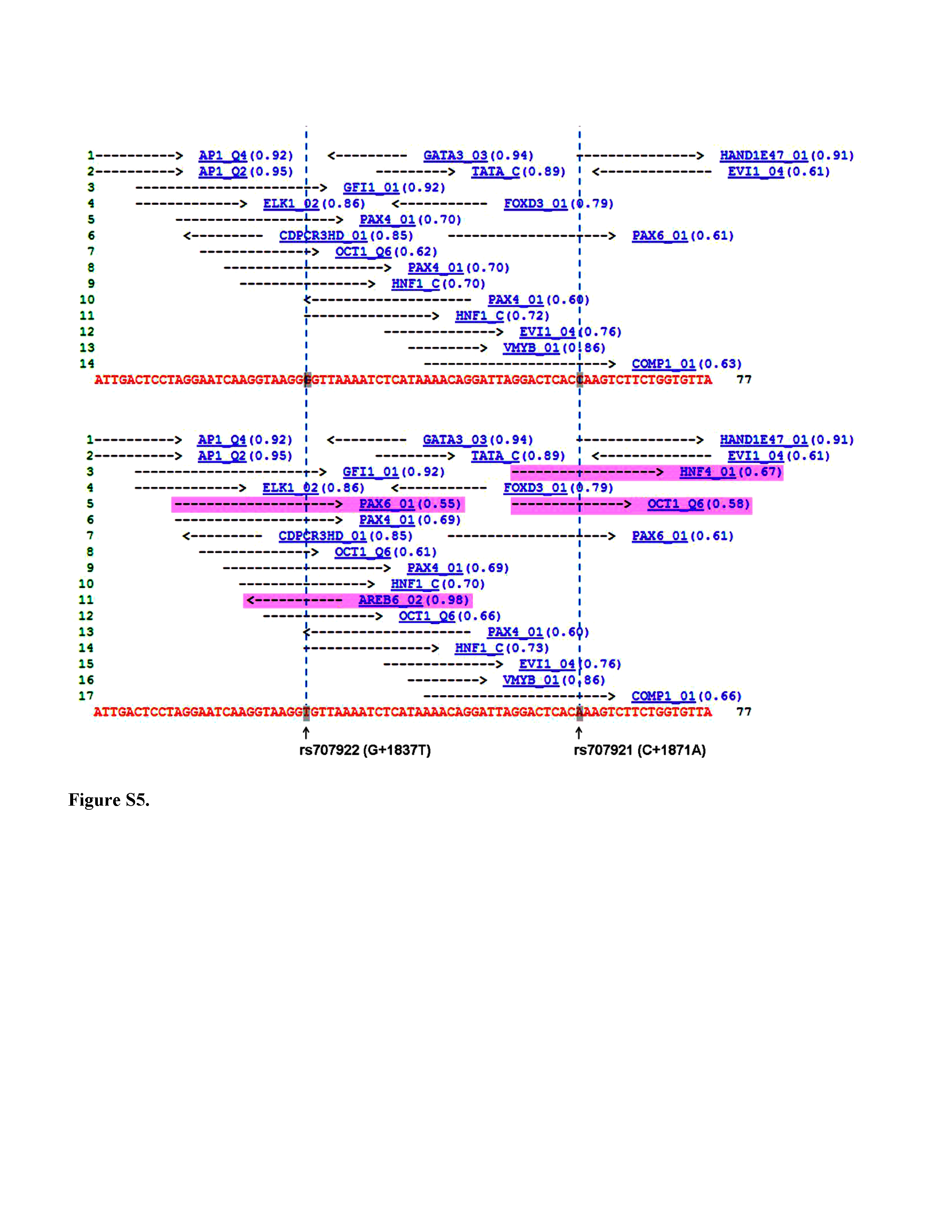

Supplement: Figure S5 — Computer-predicted transcription factor interaction sites in nucleotide sequences spanning SNPs rs707922(G+1837T) and rs707921(C+1871A). This figure is generated by the MATCH program. Top panel: The transcription factors predicted to interact with the nucleotide sequences spanning the major allele of SNPs rs707922 (G allele) and rs707921 (the C allele). Bottom panel: The transcription factors predicted to interact with the nucleotide sequences spanning the minor allele of SNPs rs707922 (the T allele) and rs707921 (the A allele). The predicted transcription factors are marked by blue text with scores of matrix match indicated in parentheses. The locations and orientations of the binding sites for these predicted transcription factors are marked by black horizontal dashed lines with arrows. Highlighted in pink boxes are allele-specific transcription factors (PAX6 and AREB6 for rs707922-T; HNF4 and OCT1 for rs707921-A) and their corresponding binding sites. The vertical dashed lines indicate the locations of SNPs rs707922 and rs707921. The precise nucleotide positions of SNP rs707922 and rs707921 are also highlighted with grey boxes in the DNA sequences represented by red colored text. (TIFF) [file pone.0017324.s005.tiff]
