# Supplementary material for: Apolipoprotein M Gene (APOM) Polymorphism Modifies Metabolic and Disease Traits in Type 2 Diabetes
Source: PLoS One. 2011 Feb 24;6(2):e17324. doi: 10.1371/journal.pone.0017324 (PMC3044746; doi:10.1371/journal.pone.0017324)
Supplement: Table S1 — Sequences of primers used in this study. (PDF) [file pone.0017324.s006.pdf]

Table S1.

Primer	Sequence
5’RACE-YY1	5’-AATGGGGTGGGGGAGAACGT3’
5’RACE-YY2	5’-GCTGATGGCGATGAATGAACACTG-3’
5’RACE-YY3	5’-CCAGAACCCATCCTGCCTCCCTC-3’
5’RACE-YY4	5’-AACCCATCCTGCCTCCCTCA-3’
5’RACE-YY5	5’-CGCGGATCCGAACGCTGCGTTTGCTGGCTTTGATG-3’
3’RACE-YY6	5’-CTGGGCGTGGATGGGAAGGAGTT-3’
3’RACE-YY7	5’-GCGAGCACAGAATTAATACGACT-3’
3’RACE-YY8	5’-GGCCGCCCTGACATGAAGACTGA-3’
3’RACE-YY9	5’-CGCGGATCCGAATTAATACGACTCACTATAGG-3’
3’RACE-YY10	5’-GGCCAGGGTTACCAGCGCTTTCT-3’
3’RACE-YY11	5’- AAGGGAAGTCCAGGGAGAAAAGCAAAG-3’
RT-PCR-YY12	5’-GTACTCGAGATGTTCCACCAAATTTGGG-3’
RT-PCR-YY13	5’-GCTAAGCTTGTTATTGGACAGCTCACAG-3’
RT-PCR-YY14	5’-GCTAAGCTTCCCTGTAACACCAGAAGAC-3’
RT-PCR-YY15	5’-GTC<u>GGTACC</u>TTCCACCAAATTTGGGCAGC-3’
RT-PCR-YY16	5’-GTC<u>GCGGCCGC</u>TCAGTTATTGGACAGCTCACAGGC-3’
RT-PCR-YY17	5’-GTC<u>GCGGCCGC</u>TCACCCTGTAACACCAGAAGACTTG-3’
RT-PCR-Tao1	5’-ATCTCTGCCCCCTCTGCTGA-3’
RT-PCR-Tao2	5’-GGATGACCTTGCCCACAGGGT-3’
T7	5’-TAATACGACTCACTATAGGG-3’
pEGFPN1-for	5’-GTCGTAACAACTCCGCCC-3’
pEGFPN1-rev	5’-GTCCAGCTCGACCAGGATG-3’
CMV-For	5’-CGCAAATGGGCGGTAGGCGTG-3’
Mutagenesis-YY18	5’-CAAGGTAAGG<u>T</u>GTTAAAATC-3’
Mutagenesis-YY19	5’-GATTTTAAC<u>A</u>CCTTACCTTG-3’
