# Supplementary material for: Apolipoprotein M Gene (APOM) Polymorphism Modifies Metabolic and Disease Traits in Type 2 Diabetes
Source: PLoS One. 2011 Feb 24;6(2):e17324. doi: 10.1371/journal.pone.0017324 (PMC3044746; doi:10.1371/journal.pone.0017324)
Supplement: Table S2 — Summary of data quality of the five successfully genotyped APOM SNPs in the full cohort (n = 1840). The clinical traits tested for IM include T2D, TG, HbA1c, FPG, TC, HDL-C, and LDL-C. (PDF) [file pone.0017324.s007.pdf]

Table S2.

	SNP location		Major/minor alleles	MAF	Call rate	*P* value							
						HWE	IM for T2D	IM for Clinical Traits in T2D					
								IM for HbA_{1c}	IM for FPG	IM for TC	IM for HDL-C	IM for TG	IM for LDL-C
rs805297	5’ flanking	-1065	C/A	0.303	95.7%	0.485	0.144	0.933	0.912	0.954	0.252	0.568	0.511
rs9404941	5’ flanking	-855	T/C	0.248	99.5%	0.951	0.726	0.205	0.421	0.966	0.891	0.746	0.833
rs805264	Intron1	203	G/A	0.196	99.0%	0.200	< 0.001	0.329	0.658	0.891	0.466	0.616	0.667
rs707922	Intron5	1837	G/T	0.204	98.8%	0.292	0.820	0.659	0.246	0.737	0.421	0.619	0.912
rs707921	Intron5	1871	C/A	0.199	98.9%	0.174	0.486	0.131	0.388	0.874	0.132	0.828	0.861
