# Supplementary material for: Apolipoprotein M Gene (APOM) Polymorphism Modifies Metabolic and Disease Traits in Type 2 Diabetes
Source: PLoS One. 2011 Feb 24;6(2):e17324. doi: 10.1371/journal.pone.0017324 (PMC3044746; doi:10.1371/journal.pone.0017324)
Supplement: Table S3 — Frequencies of common haplotypes constructed by APOM SNPs rs805297, rs904941, and rs707922. (PDF) [file pone.0017324.s008.pdf]

Table S3.

Haplotypes	Frequency		Chi square	*P* value
	Control	T2D		
A-T-G	0.307	0.301	0.082	0.774
C-C-G	0.243	0.251	0.247	0.619
C-T-G	0.235	0.249	0.898	0.343
C-T-T	0.205	0.193	0.805	0.370
