# Supplementary material for: Apolipoprotein M Gene (APOM) Polymorphism Modifies Metabolic and Disease Traits in Type 2 Diabetes
Source: PLoS One. 2011 Feb 24;6(2):e17324. doi: 10.1371/journal.pone.0017324 (PMC3044746; doi:10.1371/journal.pone.0017324)
Supplement: Table S4 — Haplotype C-T-T formed from SNPs rs805297(C-1065A), rs9404941(T-855C), and rs707922 (G+1837T) and clinical characteristics of T2D patients. Values are either number of subjects, mean ± SD, or geometric mean (95% confidence interval). p values here represent the comparisons between subgroup of homozygotes of C-T-T haplotype vs. subgroup with one C-T-T haplotype and without C-T-T haplotype (a recessive model). p values are adjusted for age, sex, BMI and disease duration in T2D. In non-diabetic controls, the p values without parentheses are adjusted for age, sex and BMI. “+/+” represent the homozygote of haplotype C-T-T, “+/−” represent the heterozygote of haplotype C-T-T, “−/−” represent the subgroup who do not have the haplotype of C-T-T. Individuals on lipid lowering medications (n = 90) were excluded for association analysis with lipid traits. * statistical significance (p<0.0125). (PDF) [file pone.0017324.s009.pdf]

Table S4.

Phenotype	T2D				Non-diabetic control			
	+/+	+/-	-/-	*p* value ++ vs (+/-,-/-)	+/+	+/-	-/-	*p* value ++ vs (+/-,-/-)
N (M/F)	16/29	141/218	319/421	0.443	19/16	77/110	179/205	0.298
AGE (years)	52.29±12.92	50.87±13.89	49.14±13.78	0.218	43.94±7.13	41.22±10.80	41.33±10.50	0.145
BMI (kg/m^2)	24.50±3.67	25.20±4.21	25.22±4.24	0.262	23.42±2.74	23.22±3.46	22.74±3.23	0.364
SBP (mmHg)	136.91±26.08	132.79±23.15	134.67±22.78	0.417	115.58±16.08	116.29±17.10	114.91±16.14	0.940
DBP (mmHg)	76.07±12.14	76.05±11.43	77.40±11.03	0.601	75.32±10.41	72.79±11.74	71.78±10.97	0.102
$HbA1_c$ (%)	8.62±2.45	7.85±1.80	7.88±1.82	0.007*				
FPG (mmol/l)	10.23±3.93	8.83±3.72	8.98±3.42	0.015	4.93±0.36	4.84±0.43	4.83±0.41	0.209
TC (mmol/l)	6.05±1.41	5.32±1.16	5.48±1.28	0.001*	5.17±0.80	4.94±1.02	5.08±0.93	0.952
HDL-C (mmol/l)	1.37±0.47	1.26±0.36	1.26±0.37	0.127	1.54±0.39	1.56±0.44	1.55±0.42	0.833
TG (mmol/l)	1.46 (0.74-2.88)	1.38 (0.72-2.66)	1.43 (0.73-2.80)	0.593	1.11 (0.60-2.02)	0.90 (0.52-1.55)	0.93 (0.54-1.60)	0.318
LDL-C (mmol/l)	3.85±1.20	3.32±1.91	3.40±1.02	0.003*	3.03±0.73	2.95±0.87	3.02±0.82	0.669
